# Clinical prognostic implications of EPB41L4A expression in multiple myeloma

**DOI:** 10.7150/jca.33805

**Published:** 2020-01-01

**Authors:** Weilong Zhang, Rui Lai, Xue He, Xiaoni Liu, Ye Zhang, Zuozhen Yang, Ping Yang, Jing Wang, Kai Hu, Xiaoliang Yuan, Xiuru Zhang, Weiyou Liu, Hongmei Jing

**Affiliations:** 1 Department of Hematology, Lymphoma Research Center, Peking University Third Hospital, Beijing, 100191, China; 2 Department of the Respiratory medicine, The People's Hospital of Ruijin City, Ruijin, 342500, China; 3 Department of Pathology, Beijing Tiantan Hospital, Capital Medical University, Beijing, 100070, China; 4 Department of Respiratory Medicine, First Affiliated Hospital Gannan Medical University, Ganzhou, 341000, China; 5 Melbourne School of Population and Global Health, The University of Melbourne, Victoria, 3010, Australia

**Keywords:** EPB41L4A, multiple myeloma, prognostic, gene expression profile.

## Abstract

**Background:** Multiple myeloma (MM) is one of the most common incurable malignancies in malignant plasma cell disease. EPB41L4A is a target gene for the Wnt/β-catenin pathway, which is closely related to the survival of multiple myeloma cells. However, there is currently no research report on the prognostic significance of the EPB41L4A gene in MM.

**Methods:** We studied the biological significance and prognostic significance of EPB41L4A expression in MM by integrating 1956 MM samples from 7 datasets, and explored the relationship between EPB41L4A expression and MM ISS stage, molecular type, therapeutic response and survival.

**Results:** We found that the expression level of EPB41L4A is inversely proportional to the copy number of 1q21 (P = 3.4e-13). EPB41L4A was low expressed in MAF, MMSET and proliferating molecular typing patients (P <= 0.001). High expression of EPB41L4A can predict good survival in MM (EFS: P < 0.0001; OS: P < 0.0001). We found that patients with relapsed MM had lower expression levels of EPB41L4A than those without recurrence (P = 0.0039). We also found that EPB41L4A can predict the prognosis of MM patients may be related to DNA replication. These results indicate that the initial expression level of EPB41L4A can predict the prognosis of MM patients.

**Conclusions:** We found that the high expression of EPB41L4A predicts good survival level in MM.

## Background

Multiple myeloma (MM) is one of the most common incurable malignancies in malignant plasma cell disease.^1^ It is characterized by malignant proliferation of monoclonal plasma cells in the bone marrow and secretion of a large number of monoclonal immunoglobulin.^2^ The main clinical manifestations were infection, anemia, immunosuppression, bone destruction and renal failure.^3^ In recent years, the incidence of MM has gradually increased; accounting for about 1% of all cancers, and about 10% of hematopoietic tumors.^4^ The median overall survival can be several months or more than ten years.^5^ At present, the specific mechanism of plasma cell disorders and progression to symptomatic MM disease has not been fully elucidated. However, there has been some progress in the study of the staging of MM disease, which can predict the prognosis of patients. In the early years, staging of tumors was performed by using the Durie-Salmon staging system.^6^ In recent years, the International Myeloma Working Group (IMWG) has proposed an international staging system (ISS) for staging multiple myeloma with β2-MG and serum albumin levels.^7^ It helps to predict the prognosis of the disease. With the development of molecular diagnostic techniques, the IMWG proposed the revised International Classification System (R-ISS) in the original ISS staging system in 2015.^8^, ^9^ The treatment options of MM vary, but at present it is chemotherapy-based and autologous stem cell transplantation (ASCT).^10^, ^11^ At present, some studies have shown that some genes can predict the clinical outcome of MM patients. For example, 1q21 amplification is the most common chromosomal mutation in MM patients and indicates a poor prognosis.^12^ And two GEP scores for MM patient classification and prognosis (based on the Arkansas GEP70 score and the HOVON SYK92 score) have been commercialized. We can still find some "new genes" that can also predict the clinical outcome of MM patients. The "new gene" can be integrated with genes that have been found to predict the prognosis of MM patients to better predict the prognosis of MM patients, and can also provide a reference for opening up a new therapeutic pathway.

EPB41L4A (*e*rythrocyte *p*rotein *b*and *4.1-l*ike *4a,* also called Nbl4) which is a member of the band FERM (Four-point-one, Ezrin, Radixin, Moesin) protein superfamily were discovered in almost all cellular organism in molecular ecology, especially high expressions in brain, liver, thymus, and peripheral blood leukocytes.^13^ The protein superfamily members are important regulators of embryonic development. They are a group of membrane-associated proteins whose major biological functions are involved in the regulation of cytoskeletal rearrangements, intracellular trafficking and signal transduction. Studies have suggested that mutations in several genes in the FERM protein family are associated with human cancers and blood cell diseases.^14^ Studies have shown that EPB41L4A is a target gene for the Wnt/β-catenin pathway and is associated with cell polarity or proliferation. (13)Beta-catenin is a key regulator of cell proliferation and is frequently mutated in various human cancer types. Recent advances in cancer research have revealed that β-catenin plays a role in intercellular adhesion and Wnt signaling pathways and is also a major player in carcinogenesis in various tissues (colon, liver, ovary, skin, and blood);^15^-^18^ the Wnt signaling pathway plays an important role in regulating cell proliferation, migration, differentiation, and stem cell self-renewal, which is closely related to the survival of multiple myeloma cells.^19^

Only a few studies have shown that EPB41L4A has genetic susceptibility to colon, nerve/brain muscle and congenital keratosis. 20 However, there is currently no clinical prognostic study on the EPB41L4A gene in any types of cancer. The study integrates multiple data from MM to achieve three goals. First, determine the clinical relationship between EPB41L4A and MM. Second, to understand whether EPB41L4A acts on MM is related to the cell cycle. Finally, understand the relationship between this gene and the recurrence before and after treatment.

## Methods

### Data source

In our study, gene expression microarrays of 1956 MM samples were derived from Gene Expression Omnibus (GEO) database. We selected patients in our research with the criteria as follows. 1) All MM patients should have the whole transcriptome gene expression data. 2) All the patients should include some clinical information such as clinical biochemical examination, therapy regimen or therapy response. GSE24080 (559 samples from 559 patients),^21^ the relationship between EPB41L4A expression and ISS stage, serotype, survival (EFS and OS), 1q21 amplification, molecular subtype, and pathway was analyzed. GSE9782 (264 samples from 264 patients),^22^ the relationship between EPB41L4A expression and survival (OS) was analyzed. GSE82307 (66 samples from 33 patients),^23^ we analyzed the relationship between EPB41L4A expression before and after relapse or treatment. GSE19554 (38 samples from 19 patients),^24^ we analyzed the relationship between EPB41L4A expression before and after treatment. GSE19784 (308 samples from 308 patients),^25^ the relationship between the expression of EPB41L4A and the 9 molecular types was analyzed. GSE83503 (585 samples from 585 patients),^26^ the relationship between expression of EPB41L4A and relapse was analyzed. GSE9782 (238 samples from 238 patients),^22^ the relationship between the expression of EPB41L4A and the drug treatment response (dexamethasone and bortezomib) was analyzed using the U133A and U133B array test methods, respectively. GSE39754 (136 samples from 136 patients),^27^ the relationship between the expression of EPB41L4A and ASCT after drug treatment induction therapeutic response was analyzed. Our research is in line with the Helsinki Declaration. And our research was approved by the Ethics Committee of Peking University Third Hospital and the Ethics Committee of Gannan Medical University. These patients have signed informed consent and obtained approval from the Ethics Committee.^21^-^27^

### Microarray analysis

Gene expression of each dataset was calculated by RMA method (robust multiarray averaging). The relative RNA expression value of each gene was log-transformed using log2. We analyzed expression of each gene and the survival (EFS and OS) using survcomp package (hazard.ratio function) with the GSE24080 datasest. Hazard ratio and P-value was estimated through Cox regression model. EPB41L4A gene is in the high rank among all genes by ranking with the P-value.

The cut point of EPB41L4A gene expression was conducted using survminer package (surv_cutpoint function) with maximally selected rank statistics method. The MM patients with higher expression than this cut point constituted the EPB41L4A high group, and the MM patients with expression lower than this cut point constituted the EPB41L4A low group. The gene expression profiles of EPB41L4A high group and EPB41L4A low group were analyzed. P < 0.05 in unpaired t test and Foldchange (FC, log2) > 0.8 or < -0.8 was used to indicate expression levels of different genes.

### Gene Ontology (GO) analysis

By using the default parameters of the DAVID tool,^28^ different expressed genes between EPB41L4A high group and EPB41L4A low group was analyzed of the pathway enrichment. The results were ranked by the P value (-log10).

### Statistics

This study performed statistical analysis by R software v3.1.3 (ggplot2 and survminer package). More than two sets of samples were compared using the Kruskal-Wallis test. The log-rank test, Cox regression analysis and Fisher's exact test are used for survival analysis. The average values of two samples were compared using the unpaired t test or Wilcoxom test. The average values of more than two samples were compared using the Anova.

## Result

### Low expression of EPB41L4A in MAF, MMSET and proliferating molecular typing

**Figure 1:**
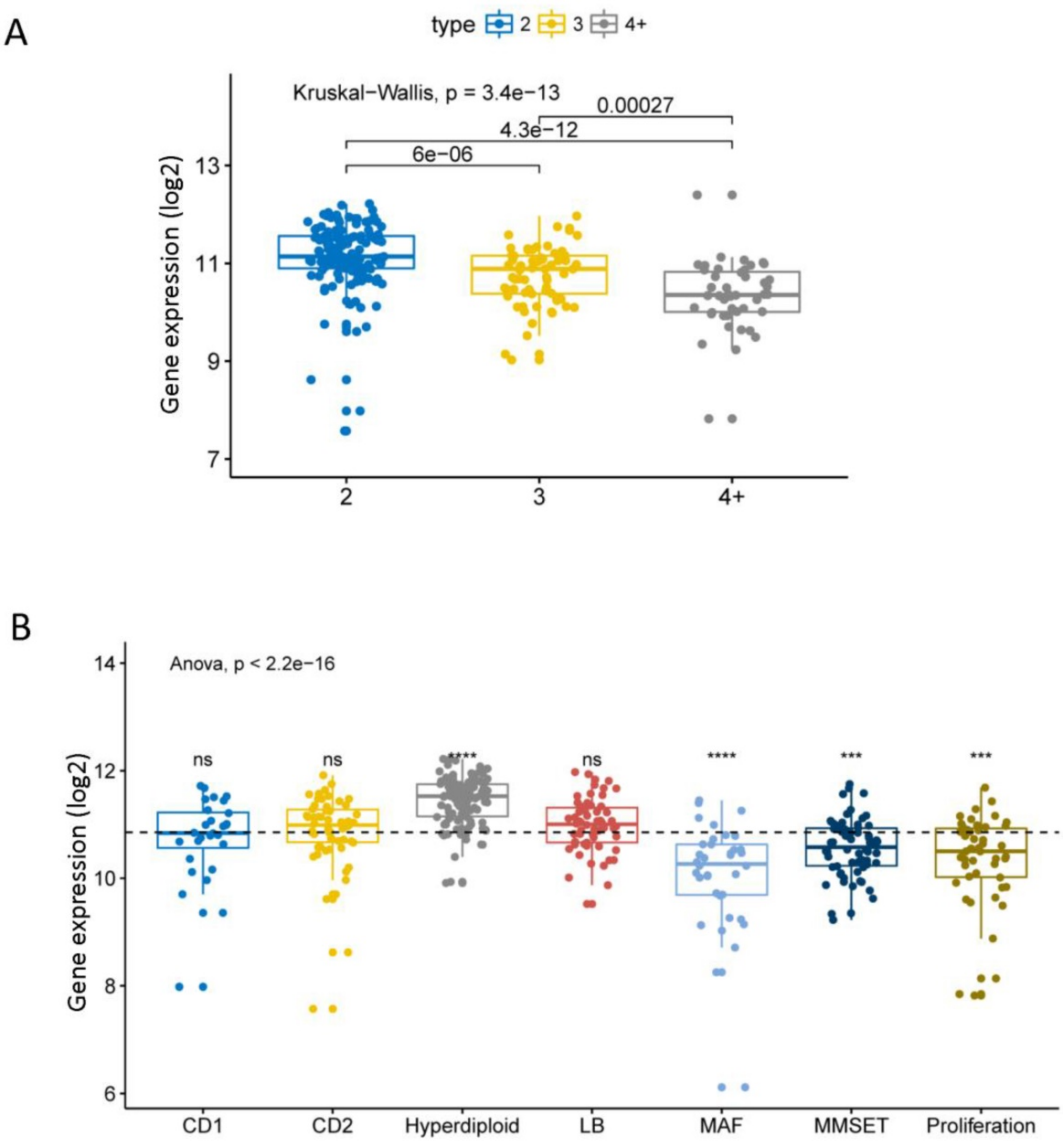
Expression level of EPB41L4A in 1q21 at different amplification levels and 7 molecular typing. Each dot represents each MM patient; Data analysis of 559 MM patients from GSE24080. A, The expression levels of EPB41L4A were compared in MM patients with different amplification levels of 1q21. The x-axis represents the 1q21 amplified copy number; the y-axis represents the EPB41L4A expression level (log2); different colors represent different 1q21 amplified copy numbers. The total P = 3.4e-13, Kruskal-Wallis test. B, The x-axis represents 7 molecular typing; the y-axis represents gene expression levels (log2); the average values of two samples were compared using the unpaired t test; the average values of more than two samples were compared using the Anova. ns: P > 0.05, *: P <= 0.05, **: P <= 0.01, ***: P <= 0.001, ****: P <= 0.0001.

We compared the expression levels of EPB41L4A at different amplification levels of 1q21. This result shows that the higher the amplification levels of 1q21, the lower the expression level of EPB41L4A gene. There was a significant difference in the expression level of the gene between each amplification level (dataset GSE24080, total 559 samples, P = 3.4e-13, Kruskal-Wallis Test, Figure 1A). The comparison of the expression level of EPB41L4A in all 7 molecular subtypes with the average value of all molecular subtypes showed that the gene was highly expressed in the hyper diploid type with statistical significance (dataset GSE24080, total 559 samples, P <= 0.0001, unpaired t test, two sided, Figure 1B). The expression level of EPB41L4A was significantly lower and statistically significant in patients with MAF, MMSET and proliferating molecular types (dataset GSE24080, total 559 samples, P <= 0.001, unpaired t test, two sided, Figure 1B). However, comparing the expression levels of EPB41L4A in CD1, CD2 and LB molecular types and the average level of all 7 molecular types was not statistically significant (dataset GSE24080, total 559 samples, P > 0.05, unpaired t test, two sided, Figure 1B). We also compared the expression levels of the 9 molecular types (the other method of molecular typing) with the average of all molecular types. The data was derived from another dataset GSE19784, a total of 308 MM specimens. The expression level of EPB41L4A in 9 molecular typing patients is approximately the same as that in 7 molecular typing (P = 2.4e-07, Anova, Figure S1 and Table S1). Similarly, the expression level of EPB41L4A was decreased in proliferative molecular type, although there was not statistical significant (P > 0.05, unpaired t test, two sided, Figure S1).

To compare the expression level of EPB41L4A gene in different stages of MM, we analyze 559 MM expression profiles from the GSE24080 dataset. There was a significant difference in the expression of EPB41L4A gene between ISS I and III phase of MM (P = 0.019, Wilcoxon Test, Figure S2A). However, comparing this gene expression level between ISS I and ISS II Phase was not statistically significant (P = 0.46, Wilcoxon Test, Figure S2A), and the comparison of gene expression levels between ISS II and ISS III was also not statistically significant (P = 0.15, Wilcoxon Test, Figure S2A). We also compared the expression levels of EPB41L4A at different stages of different serotypes. In the serotype IgA group, we found that the expression level of EPB41L4A was lower in the ISS III than in the first two stages, and the difference in the expression level of EPB41L4A in ISS II and ISS III was statistically significant. (P = 0.045, Wilcoxon Test, Figure S2B). However, there was no significant difference in the expression levels of this gene between ISS I and ISS II (P = 0.46, Wilcoxon Test, Figure S2B), and similarly no significant difference between ISS I and ISS III (P = 0.084, Wilcoxon Test, Figure S2B). The expression of EPB41L4A gene in serum-free light chain (FLC) and serotype IgG groups was not statistically significant at all Phases. (FLC: P = 0.8, IgG: P = 0.15, Kruskal-Wallis Test, Figure S2B)

### EPB41L4A expression predicts the survival level in MM

**Figure 2:**
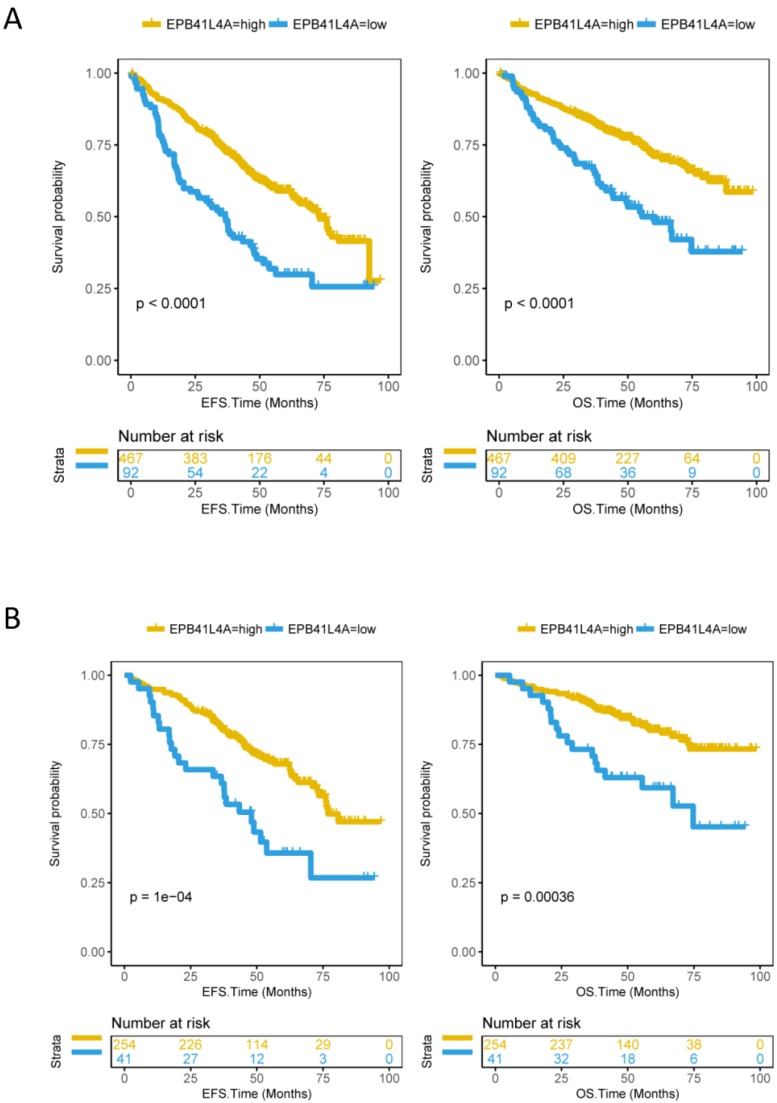
Compare the survival levels of the EPB41L4A-high group and the EPB41L4A-low group in MM patients. Left side: the x-axis represents the EFS time (months); the y-axis represents the survival probability. Right side: the x-axis represents the OS time (months); the y-axis represents the survival probability. The data was derived from the GSE24080 dataset. A, Event-free survival and overall survival in 559 MM patients (EFS: P < 0.0001; OS: P < 0.0001). To compare the survival curves of high and low gene expression, a log-rank test was used. EFS, Event-free survival time (months); OS, Overall survival time (months). B, Event-free survival and overall survival in 295 MM patients with ISS I phase (EFS: P = 1e-04; OS: P = 0.00036; log-rank test).

Since EPB41L4A is low expressed in proliferative patients, we further analyzed the relationship between the expression level of EPB41L4A and survival. We compared event-free survival (EFS) and overall survival (OS) in 559 MM patients (dataset GSE24080) with the EPB41L4A-high group and the EPB41L4A-low group. We found that the high expression of EPB41L4A predicts significantly good EFS (P < 0.0001, log-rank test, Figure 2A) and OS (P < 0.0001, log-rank test, Figure 2A). We also analyzed the relationship between EPB41L4A expression level and survival through an independent dataset (dataset GSE9782, total 264 samples). Similarly, we found that EPB41L4A-high group had better OS (P < 0.0001, log-rank test, Figure S4) than EPB41L4A-low group. In addition, we also further analyzed the expression of this gene in the early (ISS I) and mid-late (ISS II and III) of MM patients with OS and EFS. The results showed that the expression level of EPB41L4A is closely related to OS and EFS in various stages of the disease. Especially in the early stage of the disease, the expression level of the gene has more significant difference in the survival period (ISS I: EFS P = 1e-04, OS P = 0.00036; ISS II and III EFS P = 0.00048, OS P = 0.0064; log-rank test; Figure 2B and Figure S5).

### EPB41L4A expression as an independent prognostic factor in MM patient

We found that EPB41L4A was an independent prognostic factor in 559 MM patients (EPS: P = 8.80e-06, OS P = 7.54e-05, Cox regression analysis, Table S2). The EFS hazard ratio for EFS41L4A was 0.50 (95% CI: 0.37 -0.68, Cox regression analysis, Table S2) and the OS risk ratio for EFS41L4A was 0.49 (95% CI: 0.34-0.70, Cox regression analysis, Table S2). The Table S2 shows that EPB41L4A is a prognostic factor independent of β-2 microglobulin (B2M), LDH, albumin 21, MRI and bone marrow biopsy plasma cells (BMPC). In addition, to ensure that the comparison between the EPB41L4A-high group and the EPB41L4A-low group is significant and reduces bias, we excluded confounding factors. The basic clinical features of SEX, RACE and ISOTYPE of MM patients enrolled in these two groups were essentially the same (P > 0.05, Fisher's exact test, Table S3). In addition, baseline characteristics of B2M, CRP, CREAT, ALB, ASPC, BMPC, and MRI were also consistent (P > 0.05; unpaired t-test, bilateral; Table S3). However, the clinical features of LDH and hemoglobin (HGB) were inconsistent between the two groups of MM patients (LDH: P < 0.001; HGB: P = 0.025; unpaired t test, two sided; Table S3). We also analyzed the clinical features of MM patients based on EPB41L4A expression levels in other datasets, except for ISOTYPE, ALB (dataset GSE9782, P <0.001, Table S4), other clinical features (SEX, RACE, Age, B2M, treatment and treatment response) were essentially identical (P > 0.05, Table S4).

### Both passive regulation of cell division and DNA replication were meaningfully enriched pathways in EPB41L4A-high group MM

**Figure 3:**
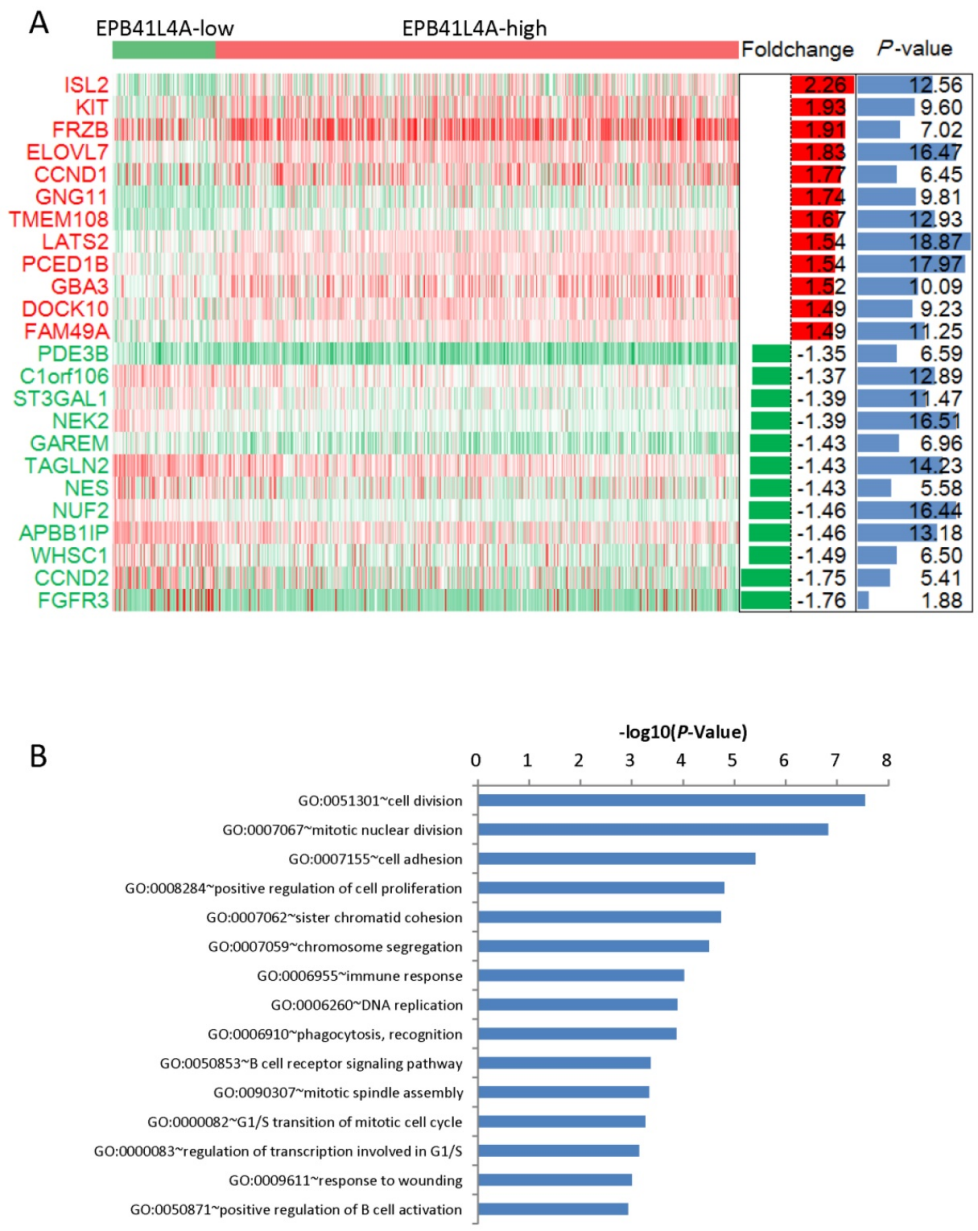
Different expression genes between the EPB41L4A-high group and EPB41L4A-low group of MM patients. A, Different expression genes between the EPB41L4A high group and EPB41L4A low group are showed by heat map. Red indicates high expression, green indicates low expression, and white indicates intermediate expression. Only display top 12 up-regulated genes and top 12 down-regulated genes. The bar graphs of foldchange (log2, left) and P values (-log10, right) match the expression levels of genes in the heat map. B, The top 15 most enriched pathways of different expressed genes from EPB41L4A-high group vs EPB41L4A-low group. The x-axis represents the P value (-log10) and the y-axis represents the top 15 pathway.

**Figure 4:**
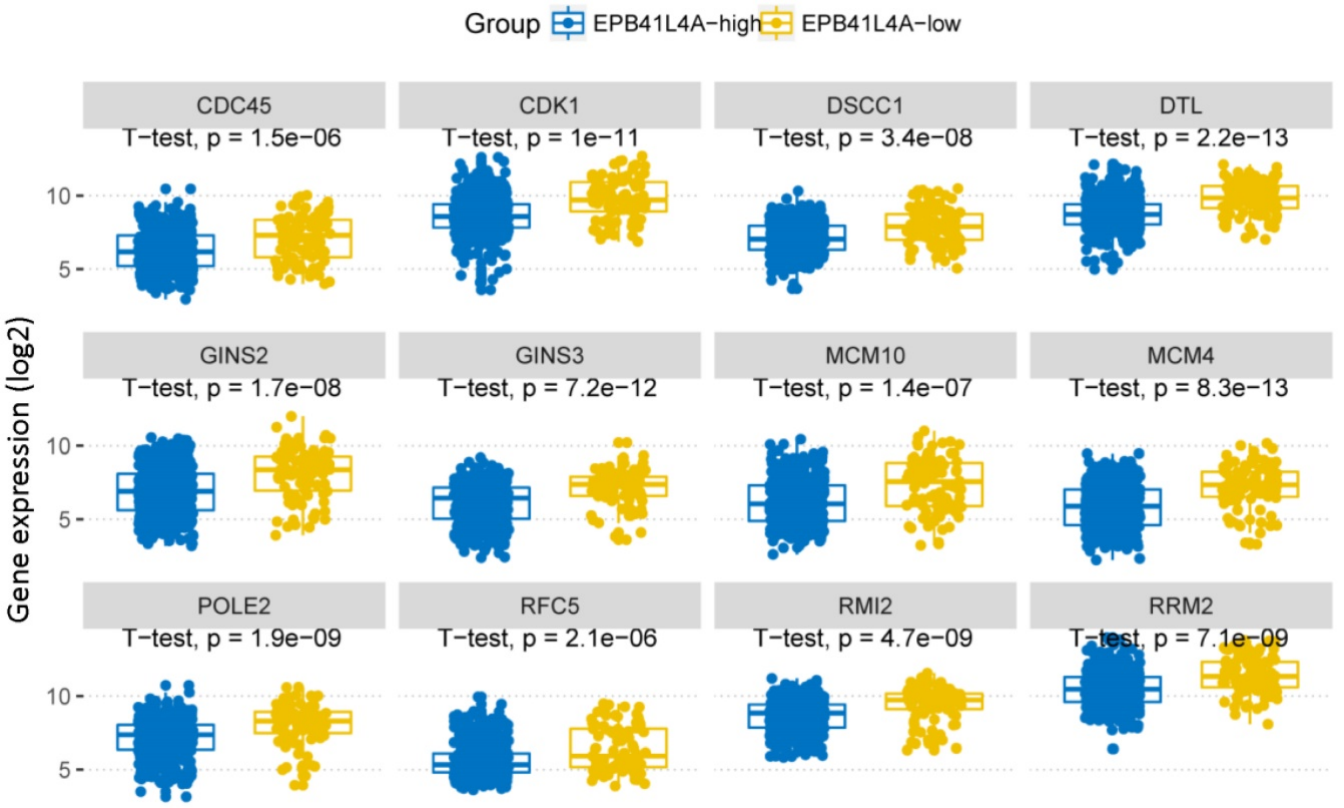
In DNA replication pathway, differently expressed genes between EPB41L4A-high group and EPB41L4A-low group in MM. GO:0006260~DNA replication, P-value (log2), unpaired t test, two sided.

We compared the gene expression profiles of the EPB41L4A-high group and the EPB41L4A-low group MM (Figure 3A). In conclusion, 227 up-regulated genes and 233 down-regulated genes were observed between EPB41L4A-high group and EPB41L4A-low group in MM. Figure 4 shows top 12 up-regulated genes and top 12 down-regulated genes. We found that ISL2 and CCND1 gene expression was up-regulated in EPB41L4A-high group (dataset GSE24080, total 559 samples, ISL2: P < 0.001; CCND1 P < 0.001; Figure 3A). However, FGFR3 and CCND2 are down-regulated genes, that is the expression of FGFR3 and CCND2 is elevated in patients with low expression of EPB41L4A (dataset GSE24080, total 559 samples, FGFR3: P < 0.001; CCND2 P < 0.001; Figure 3A). It is further shown that high expression of EPB41L4A is beneficial to prognosis. Through Gene Ontology (GO) analysis, we found that the top 15 pathway is related to the cell cycle, and the most significant enrichment pathway through all the expressed genes is cell division, mitotic nuclear division and cell adhesion (dataset GSE24080, total 559 samples, P < 0.0001, Figure 3B). We were surprised to find that in the DNA replication pathway all of the different gene expressions are down-regulated (dataset GSE24080, total 559 samples, P < 0.0001, Figure 4).

### The relationship between EPB41L4A and MM recurrence

**Figure 5:**
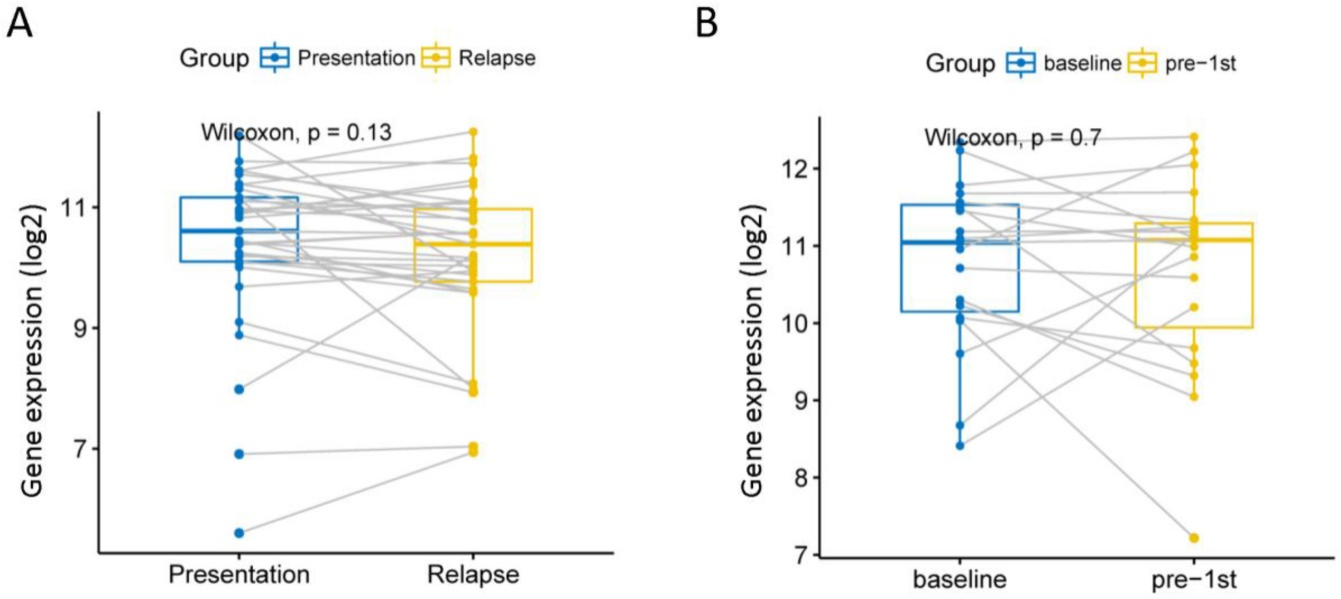
We analyzed the relationship between EPB41L4A expression before and after relapse or treatment. Gray lines are connected to samples from the same patient. A, Compare the expression levels of EPB41L4A before and after recurrence in the same patient. 33 MM patients from dataset GSE82307; P = 0.13, wilcoxon text. B, Compare the expression levels of EPB41L4A between untreated and after pre-1st chemotherapy in the same patient. 19 MM patients from dataset GSE19554; P = 0.7, wilcoxon text.

Although current high-dose therapy, autologous stem cell transplantation, chemotherapy and supportive care can significantly improve MM survival, almost all MM patients will eventually relapse, so we explore whether this gene is associated with recurrence. We analyzed 585 MM expression profiles from the GSE83503 dataset. We found that patients with relapsed MM had lower expression levels of EPB41L4A than those without recurrence (P = 0.0039, unpaired t test, two sided, Figure S3 and Table S5). We compared the expression of EPB41L4A before and after recurrence in 33 pairs of MM patient samples from the GSE82307 dataset. We found no difference in the expression of EPB41L4A before and after relapse (P = 0.13, Wilcoxon Test, Figure 5A and Table S6). We also compared EPB41L4A expression before and after treatment in 19 pairs of MM patient samples from the GSE19554 dataset. We found no difference in the expression of EPB41L4A before treatment and pre-1st (P = 0.7, Wilcoxon Test, Figure 5B).

### The relationship between EPB41L4A expression and therapeutic response

To further understand the relationship between EPB41L4A expression and therapeutic response, we compared the relationship between EPB41L4A expression and dexamethasone (Dex) and bortezomib treatment responses, respectively. We found that EPB41L4A expression was elevated and statistically significant in dexamethasone-treated partial response (PR) patients (dataset GSE9782, total 238 samples, P <= 0.05, Anova, Figure S6). However, the expression of EPB41L4A was not statistically significant in the other treatment responses of Dex and bortezomib (dataset GSE9782, 238 samples, P > 0.05, Anova, Figure S6). We also compared the relationship between the expression of EPB41L4A and the therapeutic response of autologous stem cell transplantation (ASCT) after three drug-induced treatments (vincristine, doxorubicin, and dexamethasone (VAD)). We found that EPB41L4A expression was not statistically significant in all of its therapeutic responses (dataset GSE39754, 136 samples, P > 0.05, Anova, Figure S7).

## Discussion

As the understanding of the pathogenesis of MM patients continues to improve,^29^ the heterogeneity of the disease becomes more and more obvious. These "high-risk" patients often have one or more characteristics of cytogenetic abnormalities, but these abnormalities do not always lead to poor prognosis.^30^ EPB41L4A is a key target of Wnt signaling pathway,^13^ and Wnt signaling pathway can regulate cell proliferation and differentiation,^19^ and is related to renal cell carcinoma complicated with complex of colon, nerve/brain muscle, congenital keratoses and tuberous sclerosis.^31^ It is closely related to the survival of MM patients.^19^ At present, there is no research report on prognostic significance and biological implication of EPB41L4A gene in MM. Therefore, by integrating several datasets, we found that the expression level of EPB41L4A is inversely proportional to the MM stage, and the prognosis of MM patients with high expression of EPB41L4A is better.

A number of previous studies have shown that the long non-coding RNA EPB41L4A-AS1 and the long non-coding RNA EPB41L4A-AS2 are associated with a variety of cancers. For example: 1) EPB41L4A-AS1 is a p53-regulated gene. And the consumption of EPB41L4A-AS1 in tumor treatment can increase the sensitivity of tumors to glutaminase inhibitors.^32^ 2) EPB41L4A-AS1 plays an important role in the diagnosis and prognosis evaluation of various cancers including cervical cancer, liver cancer, breast cancer, bladder cancer, glioblastoma, and colorectal cancer.^33^-^35^ That is, the low expression of EPB41L4A-AS1 is associated with a low survival rate of these cancer types. 3) EPB41L4A-AS2 is an important target for anti-metastasis treatment in patients with head and neck squamous cell carcinoma (HNSCC).^36^ It can also effectively evaluate the prognosis of anti-metastasis treatment in patients with HNSCC. 4) Long non-coding RNA EPB41L4A-AS2 can inhibit cell proliferation, invasion and promote apoptosis of hepatocellular carcinoma, renal cancer, non-small cell lung cancer and breast cancer. 37-40 However, there is currently no clinical survival study on the EPB41L4A gene in any types of cancer. There are only a few studies related to genetics of the EPB41L4A. For example: 1) Studies have revealed genetic alterations in two patients with tuberous sclerosis complex-associated renal cell carcinoma (TSC-RCC) by whole exome sequencing (WES). However, only one patient with TSC-RCC was found to have a genetic alteration in EPB41L4A.^31^ 2) It was found by WES that EPB41L4A has genetic susceptibility to colon, nerve/brain muscle and congenital keratosis.^20^ To understand the role of EPB41L4A in cancer, studies have explored the role of EPB41L4A in cell lines. This study found that when β-catenin was depleted in SW480 cells, the RNA expression of EPB41L4A was significantly reduced (SW480 cells are colorectal cancer cell lines).^41^ To the best of our knowledge, we have not found any study about the clinical prognostic relationship between EPB41L4A and tumor. In our study, we obtained 1956 samples by integrating 7 datasets and found a relationship between the expression level of EPB41L4A and the clinical prognosis of MM.

By analyzing the dataset of 559 MM patients, we found that EPB41L4A is a "good" gene, and high expression of EPB41L4A suggests a better prognosis. There are 4 points of support for EPB41L4A low expression suggest poor prognosis. 1) 1q21 gene amplification suggests a low survival rate and a poor prognosis, and the higher the amplification, the worse the prognosis.^42^, ^43^ We found that the expression level of EPB41L4A is opposite to the 1q21 amplification copy number. 2) The molecular typing by MAF, MMSET, and Proliferation suggested that MM had a poor prognosis.^44^ We found that the expression of EPB41L4A in these molecular typing was significantly lower than the average of all 7 molecular typing. 3) EFS and OS in EPB41L4A high expression group were significantly higher than EPB41L4A low expression group. 4) The expression level of EPB41L4A in patients with relapsed MM is lower than that in patients without recurrence.

Our study found that some "good" genes are also highly expressed in patients with high expression of EPB41L4A, while some "bad" genes are also highly expressed in patients with low expression of EPB41L4A. For example, these genes are ISL2, KIT, CCND1 (highly expressed in patients with high expression of EPB41L4A), CCND2 and FGFR3 (highly expressed in patients with low expression of EPB41L4A) and the like. KIT (CD117) is a transmembrane glycoprotein of the class III receptor tyrosine kinase family.^45^ Several studies have shown that c-Kit expression in MM cells is functional and associated with survival pathways (Akt pathway). High expression of KIT suggests a good prognosis.^46^-^48^ High expression of CCND1 can make MM patients have a better prognosis by highly specific inhibition of translation of myeloma cells.^49^, ^50^ However, CCND1 is also involved in the most common translocation in myeloma 11; 14. The translocation 11; 14 placed CCND1 under the transcriptional control of the immunoglobulin heavy chain (IgH) enhancer, resulting in a dysregulation of CCND1, thereby accelerating the G1 to S phase transition in the plasma cell.^51^, ^52^ Arjun Lakshman et al. found that patients with high expression of CCND1 with 11; 14 translocation had a poor prognosis.^53^ Overexpression of CCND2 reduces survival of MM patients by affecting RNA transcription.^54^, ^55^ Fibroblast growth factor receptor 3 (FGFR3) is a transmembrane tyrosine kinase receptor.^56^, ^57^ It is involved in intracellular signaling pathways and promotes cell proliferation, migration and differentiation. Overexpression of FGFR3 can lead to proliferation of myeloma cells leading to poor prognosis in MM patients, but it is often used as a therapeutic target to improve MM prognosis.^58^, ^59^ Not only is the expression of EPB41L4A highly consistent with “good” or “bad” genes in MM, but EPB41L4A expression is an independent prognostic factor for MM patients (see results).

EPB41L4A is a key target of the wnt signaling pathway,^13^ which can affect cell proliferation, migration and differentiation.^19^ EPB41L4A improves the prognosis of patients with MM and is related to the cell cycle. We found that the top 15 pathways of EPB41L4A are related to the cell cycle, and the high expression of EPB41L4A will reduce the expression of most genes in DNA replication and cell proliferation pathway. We compared the expression of EPB41L4A before and after relapse in the same MM patient and compared the expression of EPB41L4A in the same patient after untreated and pre-1st chemotherapy, and found that there was no difference in the expression of EPB41L4A before and after, but we also found that MM patients with relapsed had lower expression levels of EPB41L4A than MM patients with non-relapsed. Therefore, we hypothesize that the prognosis and recurrence levels of MM patients may be determined by the initial state of EPB41L4A expression level, but this requires further experiments to verify.

However, our research has some shortcomings. The detailed mechanism of EPB41L4A in MM is not deep enough conducted. Multiple markers were not combined to observe clinical predictions of MM patients. There is also a lack of prospective studies to demonstrate whether the expression level of EPB41L4A can alter clinical decisions for early treatment.

In summary, the high expression of EPB41L4A predicts good survival level in MM. The high expression of EPB41L4A was show as a good classifier in MM. The expression level of the EPB41L4A can predict the prognosis of MM patients, but is determined by the initial expression state of EPB41L4A, and the expression level of EPB41L4A does not change due to treatment and relapse. The EPB41L4A may be related to cell division and DNA replication pathway which can cause the better survival level and lower recurrence level of MM.

## Supplementary Material

Supplementary figures and tables.

## Abbreviations

MM: Multiple myeloma
IMWG: International Myeloma Working Group
ISS: International Staging System
R-ISS: Revised International Classification System
ASCT: autologous stem cell transplantation
EPB41L4A: erythrocyte protein band 4.1-like 4a, also called Nbl4
FERM: Four-point-one, Ezrin, Radixin, Moesin
GEO: Gene Expression Omnibus
EFS: Event-free survival time, defined from date of registration to the occurrence of death from any cause, disease progression or relapse, or censored at the date of last contact
OS: Overall survival time, defined from date of registration to the date of death from any cause or censored at the date of last contact
B2M: Beta-2 microglobulin
ALB: Albumin
HGB: Haemoglobin
MRI: Number of magnetic resonance imaging (MRI)-defined focal lesions (skull, spine, pelvis)
BMPC: Bone marrow biopsy plasma cells (%)
HR: Hazard ratio
CI: Confidence interval
CRP: C-reactive protein
CREAT: Creatinine
ASPC: Aspirate plasma cells (%)
HNSCC: head and neck squamous cell carcinoma
TSC-RCC: tuberous sclerosis complex-associated renal cell carcinoma
WES: whole exome sequencing
