# Supplementary material for: Clinical prognostic implications of EPB41L4A expression in multiple myeloma
Source: J Cancer. 2020 Jan 1;11(3):619–29. doi: 10.7150/jca.33805 (PMC6959044; doi:10.7150/jca.33805)
Supplement: Supplementary file 1 — Supplementary figures and tables. [file jcav11p0619s1.pdf]

Table S1 Cox regression analysis showed that EPB41L4A expression was an independent prognostic factor in 559 patients with multiple myeloma

		95% CI for HR		
	HR	Lower	Upper	*P*-value
EFS				
B2M (>= 3.5 mg/l)	1.44	1.05	1.97	2.21E-02
ALB (>= 35 g/l)	0.85	0.60	1.20	3.52E-01
HGB (>= 100 g/l)	0.81	0.60	1.10	1.85E-01
MRI (>= 3 focal lesions)	1.44	1.10	1.89	8.53E-03
BMPC (>= 35%)	1.46	1.06	2.01	2.17E-02
EPB41L4A (>=10.33)	0.50	0.37	0.68	8.80E-06
OS				
B2M (>= 3.5 mg/l)	1.68	1.15	2.47	7.98E-03
ALB (>= 35 g/l)	0.70	0.47	1.03	7.02E-02
HGB (>= 100 g/l)	0.91	0.63	1.30	5.95E-01
MRI (>= 3 focal lesions)	1.92	1.37	2.69	1.72E-04
BMPC (>= 35%)	1.39	0.93	2.07	1.07E-01
EPB41L4A (>=10.33)	0.49	0.34	0.70	7.54E-05

EFS: Event-free survival time (months), EFS refers to the time from the date of registration to the date of death caused by any cause, disease progression or recurrence, or the date on the date of the last contact; OS: Overall survival time (months), OS refers to the time from the date of registration to the date of death caused by any cause, or the date on the date of the last contact. HR: hazard ratio; CI: confidence interval. BMPC: Bone marrow biopsy plasma cells (%); MRI: Number of magnetic resonance imaging (MRI)-defined focal lesions (skull, spine, pelvis); HGB: Haemoglobin, g/l; ALB: Albumin, g/l; B2M: Beta-2 microglobulin, mg/l.

Table S2 Baseline characteristics of MM patients based on the expression level of EPB41L4A

		EPB41L4A-low	EPB41L4A-high	*P*-value
n		92	467	
AGE (mean (sd))		56.91 (10.59)	57.23 (9.23)	0.767
SEX (%)	female	36 (39.1)	186 (39.8)	0.993
	male	56 (60.9)	281 (60.2)	
RACE (%)	other	10 (10.9)	52 (11.1)	1
	white	82 (89.1)	415 (88.9)	
ISOTYPE (%)	FLC	13 (14.6)	71 (15.8)	0.19
	IgA	30 (33.7)	103 (22.9)	
	IgD	0 (0.0)	3 (0.7)	
	IgG	46 (51.7)	267 (59.3)	
	Nonsecretory	0 (0.0)	6 (1.3)	
B2M (mean (sd))		5.49 (6.23)	4.58 (5.18)	0.14
CRP (mean (sd))		15.22 (26.59)	10.93 (22.19)	0.104
CREAT (mean (sd))		1.54 (1.71)	1.28 (1.16)	0.069
LDH (mean (sd))		207.24 (99.88)	165.03 (54.44)	<0.001
ALB (mean (sd))		3.95 (0.55)	4.07 (0.59)	0.069
HGB (mean (sd))		10.87 (1.92)	11.33 (1.78)	0.025
ASPC (mean (sd))		44.43 (27.17)	42.35 (23.78)	0.476
BMPC (mean (sd))		51.02 (28.50)	45.48 (25.75)	0.069
MRI (mean (sd))		13.13 (16.49)	10.63 (14.11)	0.147

n: number of patients; ASPC: Aspirate plasma cells (%); CRP: C-reactive protein, mg/l; CREAT, Creatinine, mg/dl; LDH: lactate dehydrogenase, U/L; ALB: Albumin, g/l; MRI: Number of magnetic resonance imaging (MRI)-defined focal lesions (skull, spine, pelvis); HGB: Haemoglobin, g/l; BMPC: Bone marrow biopsy plasma cells (%). mean (sd): use unpaired t test, two sided; SEX (%), RACE (%) and ISOTYPE (%) use Fisher's exact test.

Fig. S1

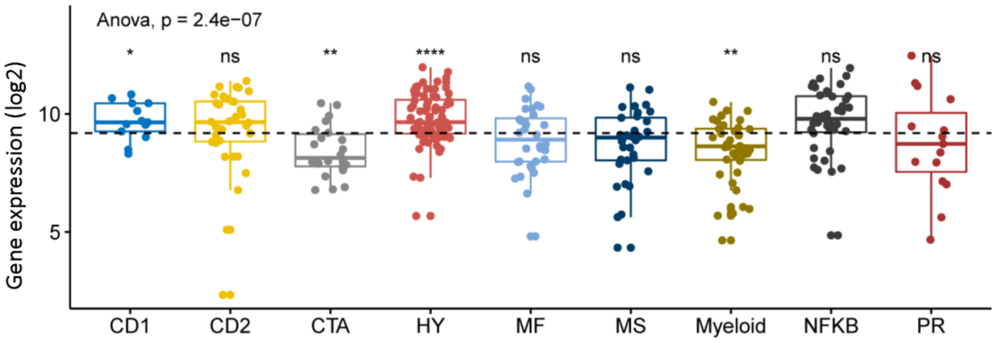
Anova, p = 2.4e−07
*
ns
**
****
ns
ns
**
ns
ns
Gene expression (log2)
10
5
CD1
CD2
CTA
HY
MF
MS
Myeloid
NFKB
PR

Fig. S2

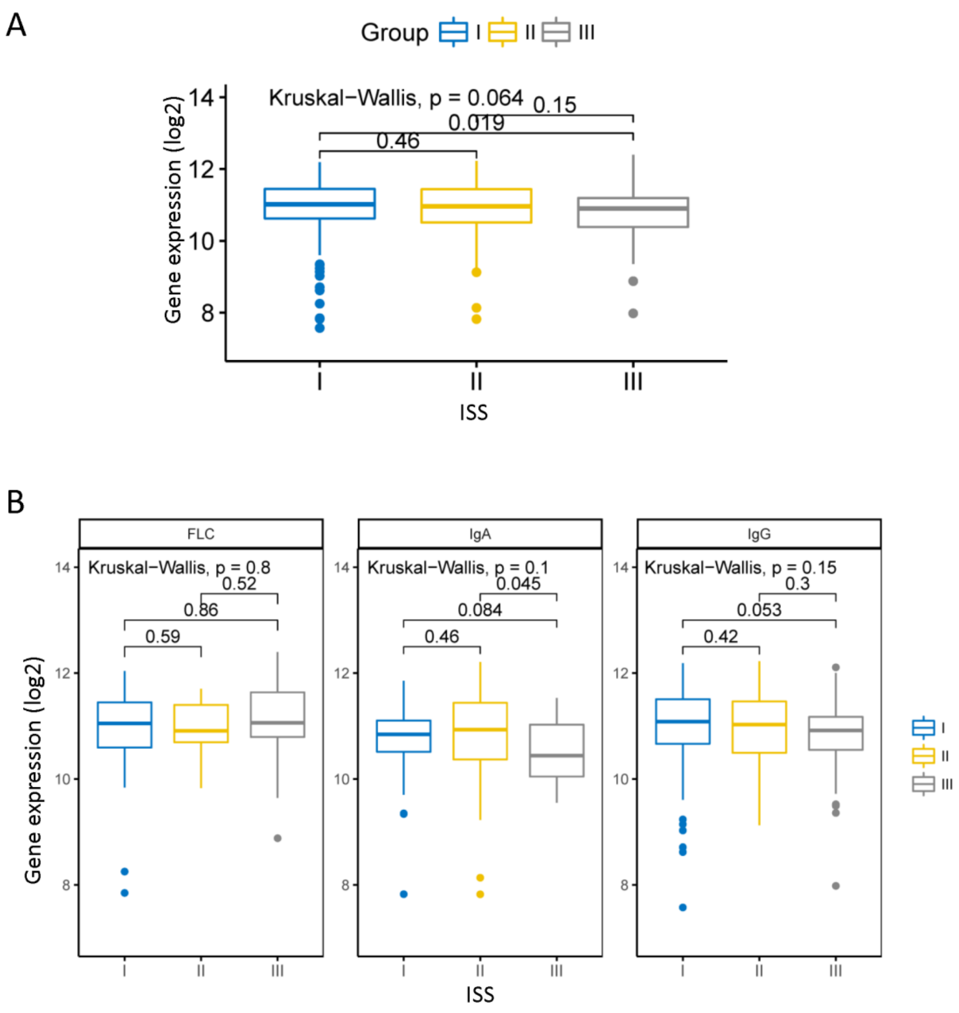

A
Group
I
II
III
Kruskal−Wallis, p = 0.064
0.15
0.019
0.46
Gene expression (log2)
14
12
10
8
I
II
III
ISS
B
FLC
IgA
IgG
Kruskal−Wallis, p = 0.8
0.52
0.86
0.59
Kruskal−Wallis, p = 0.1
0.045
0.084
0.46
Kruskal−Wallis, p = 0.15
0.3
0.053
0.42
Gene expression (log2)
ISS

Fig. S3

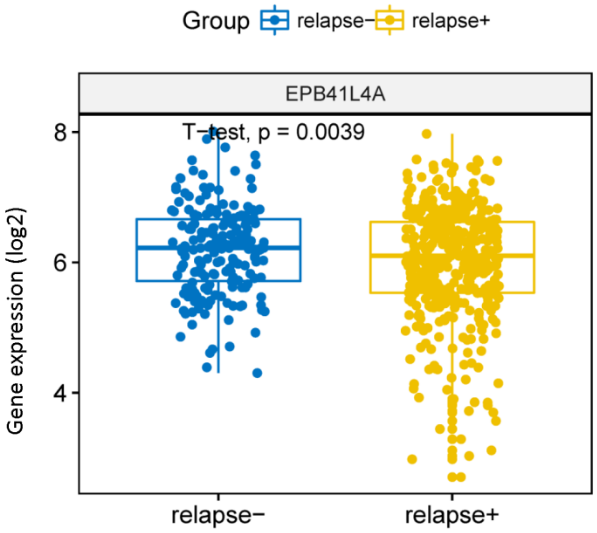
Group
relapse−
relapse+
EPB41L4A
T−test, p = 0.0039
Gene expression (log2)
8
6
4
relapse−
relapse+

Fig. S4

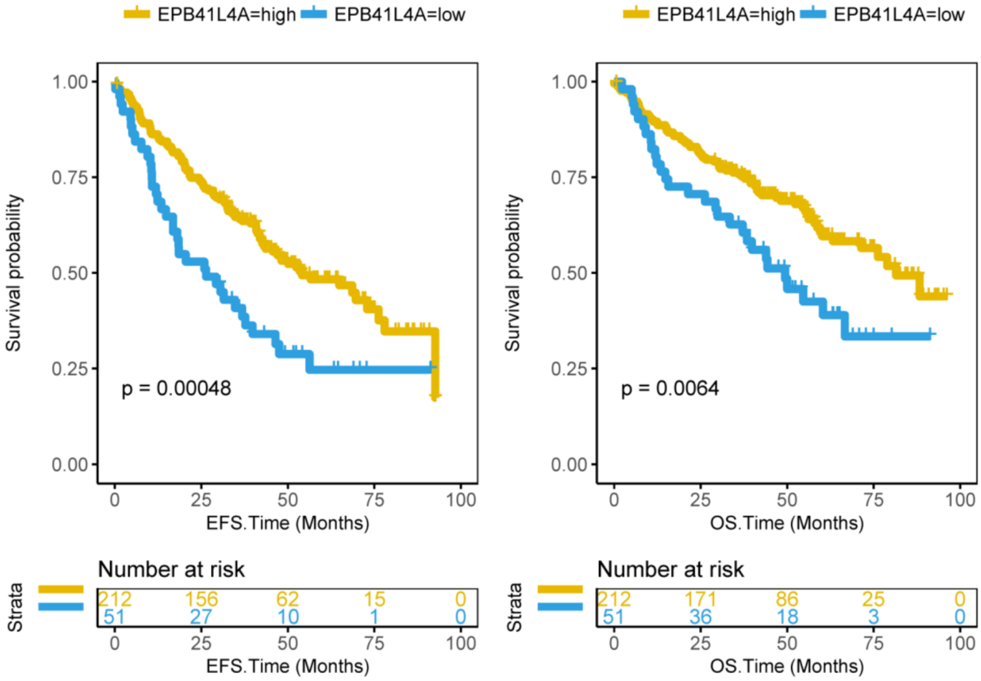

EPB41L4A=high
EPB41L4A=low
Survival probability
1.00
0.75
0.50
0.25
0.00
p = 0.00048
0
25
50
75
100
EFS.Time (Months)
Number at risk
Strata
212 156 62 15 0
51 27 10 1 0
EFS.Time (Months)
EPB41L4A=high
EPB41L4A=low
Survival probability
p = 0.0064
OS.Time (Months)
Number at risk
Strata
212 171 86 25 0
51 36 18 3 0
OS.Time (Months)

Fig. S5

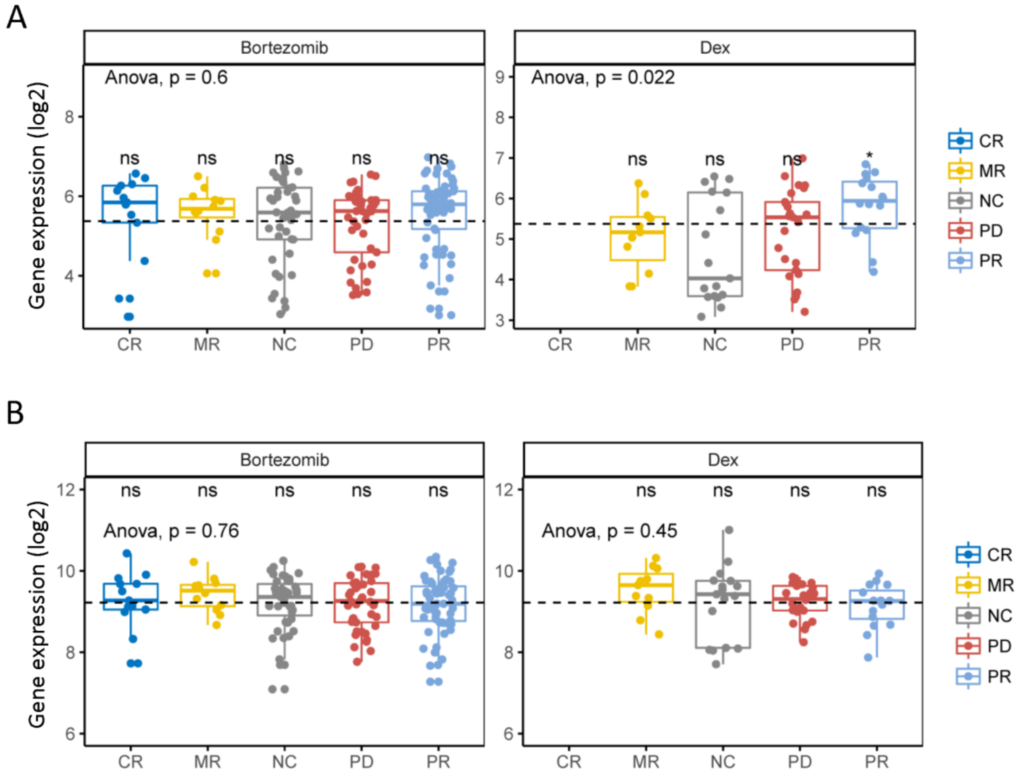
A
Bortezomib
Dex
Anova, p = 0.6
Anova, p = 0.022
Gene expression (log2)
ns
*
CR
MR
NC
PD
PR
B
Anova, p = 0.76
Anova, p = 0.45

Fig. S6

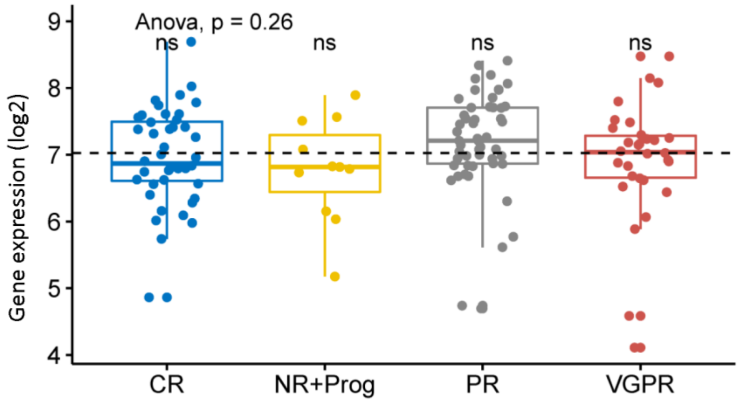
Anova, p = 0.26
ns
ns
ns
ns
Gene expression (log2)
9
8
7
6
5
4
CR
NR+Prog
PR
VGPR

Supplementary Figure Legends

Figure S1.

The expression level of EPB41L4A in 9 molecular typing mm was compared. The expression level of EPB41L4A in each molecular typing was compared with the average of the expression levels of EPB41L4A in all 9 molecular types. The x-axis represents the ISS stage; the y-axis represents the EPB41L4A expression level (log2); each point represents every MM patient; the dotted line indicates the average of the expression levels of all molecular typing of EPB41L4A. HY: Hyperdiploid molecular typing; MF: MAF molecular typing; MS: MMSET molecular typing; PR: proliferation molecular typing. The average values of two samples were compared using the unpaired t test; the average values of more than two samples were compared using the Anova. ns: P > 0.05, *: P <= 0.05, **: P <= 0.01, ***: P <= 0.001, ****: P <= 0.0001. Data were obtained from 308 MM patients in the GSE19784 dataset.

Figure S2.

Box plot of EPB41L4A expression level in 559 MM patients. The x-axis represents the ISS stage; the y-axis represents the EPB41L4A expression level (log2); the value on the line in the graph indicates the P value of the gene expression comparison at both ends of the line; More than two sets of samples were compared using the Kruskal-Wallis test; The average values of two samples were compared using the Wilcoxom test. Data analysis of 559 MM patients from GSE24080. A, Comparison of the expression levels of EPB41L4A gene in different ISS stages. B, Comparison of expression levels of EPB41L4A gene in different ISS stages of different serotypes. FLC: Serum free light chain (FLC); IgA: Serum immunoglobulin A (IgA); IgG: Serum immunoglobulin G (IgG).

Figure S3.

The expression level of EPB41L4A was compared before and after relapse in MM patients. The x-axis represents no recurrence and recurrence; the y-axis represents the EPB41L4A expression level (log2); the sample was from 585 MM patients in dataset GSE83503. P =

0.0039, unpaired t test, two sided.

Figure S4.

The survival levels of the EPB41L4A-high group and the EPB41L4A-low group in MM patients with ISS II or ISS III were compared (EFS: P = 0.00048; OS: P = 0.0064; log-rank test). Left side: the x-axis represents the EFS time (months); the y-axis represents the survival probability; Right side: the x-axis represents the OS time (months); the y-axis represents the survival probability; the data comes from the GSE24080 dataset.

Figure S5.

The relationship between EPB41L4A gene expression and drug treatment response was compared. The left panel shows that we compared the relationship between EPB41L4A expression and bortezomib treatment response. The right panel shows that we compared the relationship between EPB41L4A expression and dexamethasone (Dex) treatment response. A and B show the relationship between EPB41L4A expression and dexamethasone (Dex) and bortezomib treatment responses using U133A and U133B arrays, respectively. The samples were from 238 MM patients in dataset GSE9782. The dotted line indicates the average value of the gene expression level in each treatment response. Dex: dexamethasone; CR: Complete remission, PR: partial remission, MR: minimal remission, NC: no change, PD: progressive disease. The average values of two samples were compared using the unpaired t test; the average values of more than two samples were compared using the Anova. ns: P > 0.05, *: P <= 0.05.

Figure S6.

The relationship between the expression of EPB41L4A and the therapeutic response of autologous stem cell transplantation (ASCT) after three drug-induced treatments (vincristine, doxorubicin, and dexamethasone (VAD)) was compared. The dotted line indicates the average

value of the gene expression level in each treatment response. CR: Complete Response; VGPR: Very Good Partial Response; PR: Partial Response; NR: No Response, Stable disease; Prog: No Response, Progressive disease. The samples were from 136 MM patients in dataset GSE39754. The average values of two samples were compared using the unpaired t test; the average values of more than two samples were compared using the Anova. ns: $P > 0.05$.
